# Predictive ability of genome-assisted statistical models under various forms of gene action

**DOI:** 10.1038/s41598-018-30089-2

**Published:** 2018-08-17

**Authors:** Mehdi Momen, Ahmad Ayatollahi Mehrgardi, Ayyub Sheikhi, Andreas Kranis, Llibertat Tusell, Gota Morota, Guilherme J. M. Rosa, Daniel Gianola

**Affiliations:** 1 Department of Animal Science, University College of Agriculture, Shahid Bahonar University of Kerman (SBUK), Kerman, Iran; 2 Department of Statistical Science, University College of Mathematic and Statistical Science, Shahid Bahonar University of Kerman (SBUK), Kerman, Iran; 3 Roslin Institute, University of Edinburgh, Edinburgh, EH25 9PS UK; 4 INRA UMR1388/INPT ENSAT/INPT ENVT GenPhySE, F-31326 Castanet-Tolosan, France; 5 Department of Animal Science, University of Nebraska-Lincoln, Lincoln, Nebraska USA; 6 Department of Animal Sciences, University of Wisconsin, Madison, WI USA; 7 Department of Biostatistics and Medical Informatics, University of Wisconsin, Madison, WI USA; 8 Department of Dairy Science, University of Wisconsin, Madison, WI USA

## Abstract

Recent work has suggested that the performance of prediction models for complex traits may depend on the architecture of the target traits. Here we compared several prediction models with respect to their ability of predicting phenotypes under various statistical architectures of gene action: (1) purely additive, (2) additive and dominance, (3) additive, dominance, and two-locus epistasis, and (4) purely epistatic settings. Simulation and a real chicken dataset were used. Fourteen prediction models were compared: BayesA, BayesB, BayesC, Bayesian LASSO, Bayesian ridge regression, elastic net, genomic best linear unbiased prediction, a Gaussian process, LASSO, random forests, reproducing kernel Hilbert spaces regression, ridge regression (best linear unbiased prediction), relevance vector machines, and support vector machines. When the trait was under additive gene action, the parametric prediction models outperformed non-parametric ones. Conversely, when the trait was under epistatic gene action, the non-parametric prediction models provided more accurate predictions. Thus, prediction models must be selected according to the most probably underlying architecture of traits. In the chicken dataset examined, most models had similar prediction performance. Our results corroborate the view that there is no universally best prediction models, and that the development of robust prediction models is an important research objective.

## Introduction

The effectiveness of genomic prediction depends on the accuracy of estimation of the genetic value of individuals with yet-to-be observed phenotypes^1^. Various factors affect the accuracy of estimated genomic breeding values (GEBVs) and, hence the expected response to genomic selection. These include the model performance, training and testing sample sizes, relatedness between individuals in training and testing sets, marker density, and the statistical genetic architecture of target traits, i.e., the extent and distribution of linkage disequilibrium between markers and quantitative trait loci (QTL), number of QTLs, allelic frequencies and magnitude of QTL effects, and trait heritability^2,3^. Accuracy may vary among genomic prediction models because of different assumptions and treatments of marker effects and mode^1^. The choice of whether to use variable selection or penalized models in parametric and non-parametric contexts often depends on the typically unknown genetic architecture and heritability of the trait, as well as on sample size^4,5^. Genetic architecture is a term used to denote genotype-phenotype relationships that include the loci contributing to phenotypic variation, e.g., number of loci and their genomic location, number of alleles per locus, magnitude of their effects, pleiotropy patterns, mode of gene action and epigenetic effects^6,7^. Since statistical prediction models are used to represent unknown complexity, the term “statistical genetic architecture” may be a better term as such models cannot be taken as mechanistic representation of “genetic architecture”.

In animal and plant breeding, traits that are relevant for breeding programs have different genetic architectures. For instance, Hayes *et al*.^8^, studied three traits with presumably different underlying genetic architecture: proportion of black coat color, fat percentage, and overall type in Holstein cattle. They concluded that the models with a different variance per SNP (BayesA) were better for prediction of two of the traits that were affected by major genes; Gianola *et al*.^9^ showed that BayesA, actually assigns the same variance to each marker effect. A study by Ober *et al*.^2^ found that genomic best linear unbiased prediction (GBLUP) performed well for traits with a mostly additive genetic background (in *Drosophila melanogaster*), and conjectured an underlying epistatic gene-action when observing a poor predictive ability. In host plant resistance to wheat rust, a trait possibly influenced by additive gene effects, the Bayesian least absolute shrinkage and selection operator (BL) and ridge regression models outperformed support vector regression (SVM)^10^. Ornella *et al*.^11^, compared eleven genomic prediction models using wheat, maize, and barley data. Except for SVM, all prediction models provided similar average prediction accuracies. Howard *et al*.^12^ compared 14 genomic prediction models with 2000 biallelic markers by simulating two complex traits (explaining either 30% or 70% of the phenotypic variability) in a F2 and a backcross (BC) populations derived from crosses of inbred lines. They concluded that the parametric models predicted phenotypic values worse than those of non-parametric models when the gene action was epistasis.

The preceding suggests that the performance of genomic prediction models depends on the genetic architecture of the trait, especially major genes. Hill *et al*.^13^ and Mäki-Tanila and Hill^14^ have given strong empirical and theoretical evidence that most of the genetic variance is additive even when gene action is not. Unfortunately, the genetic architecture of most complex traits remains unknown for animal breeders and evolutionary geneticists, so a search for robust and stable prediction models is important.

The objective of this study was to compare predictive accuracy of several parametric and non-parametric genomic prediction models for quantitative traits simulated under various forms of gene actions (additive, additive-dominance, additive-dominance-epistasis and pure epistasis). Predictive accuracy of the all models was also assessed with a real chicken dataset.

## Methods

Real and simulated genomic data were used to investigate sensitivity and predictive ability of various genomic prediction models. Real data offer the advantage of reflecting true complexity, whereas simulation allows ones to explore the impact on predictive performance of factors such as statistical genetic architecture of the trait, number of markers used for the analysis, and degree of relatedness between training and prediction populations^4^.

### Simulated data

#### Population

We used a mutation–drift model with an effective population size of 100 individuals. The simulated population evolved at random for 2,000 historical generations with a constant size of 1,000 individuals per generation. To create linkage disequilibrium and to establish mutation-drift equilibrium in the historical population, a population bottleneck was introduced by decreasing population size from 1,000 to 200 at generations 1,200–1,400. Then, the historical population size was extended to 1,000 individuals for the next 800 generations^15^. A total of 400 females and 20 males from the last generation of the historical population became founders of the most recent generations. The population was then expanded in the subsequent 55 generations under random mating, each mating producing two progenies. The final 50th to 55th generations comprised of 4,800 genotyped and phenotyped animals that were used to evaluate the different prediction models.

#### Genome

The simulated genome consisted of five pairs of autosomes with 100 cM length each, leading to a 500 cM genome. At the onset, all loci were homozygous but subsequently, alleles were randomly mutated and recombined such that each loci had a mutation rate at QTLs and SNP markers of $2.5\times {10}^{-5}$ per generation. The SNP markers were randomly distributed across the genome and the initial number of markers was chosen such that it would generate a 10,000 SNP density panel of segregating bi-allelic loci with a minor allele frequency (MAF) ≥ 0.1. A total of 300 bi-allelic QTLs was simulated, whose positions were randomly distributed across the genome.

#### Simulation of phenotypes under various gene action models

Additive, dominance, and two-locus epistatic effects (i.e., additive × additive, additive × dominance and dominance × dominance interactions) were simulated in order to measure the predictive ability of various statistical prediction models. Four scenarios of gene action were simulated: additive, additive plus dominance, additive plus dominance plus epistasis, and a purely epistatic model.

#### Purely additive (Ad)

The average effect of allelic substitution measures the expected change in average phenotype produced by substituting a single allele of one type with that of another type (Table 1). This is shown as $\alpha =a+d(q-p)$, where $a$ and $d$ are additive and dominance effects, respectively, and $p$ is the allelic frequency with $q=1-p$. In previous simulation studies^16^, additive allelic substitution effects at QTLs were drawn from a Gamma distribution with parameters shown in Table 2. The effect sign was sampled to be positive or negative, each with probability 0.5. Three hundred QTLs positions were sampled from the SNPs in order to produce a purely additive trait (in this case, the dominance effect was ${d}_{ik}=0$; *i* and *k* denote the *i*-th individual and *k*-th QTL, respectively). The phenotypic value of each individual *i*, was created by adding a normally distributed residual ${e}_{i}, \sim {\rm{N}}(0,{\sigma }^{2})$ to the sum over QTL of genetic values shown in Table 1:

$${y}_{i}={\sum }_{k=1}^{nQTL}{X}_{ik}{a}_{k}+{e}_{i}$$

Above, ${X}_{ik}$ is an (*i* = 1, …, number of individuals; *k* = 1, …, number of QTLs) is an element of the incidence matrix for additive genetic effects (${a}_{k})\,\,$with 2, 1 and 0 as entries for ${A}_{2}{A}_{2}$,$\,{A}_{2}{A}_{1},\,\,$and ${A}_{1}{A}_{1}$ genotypes, respectively.

**Table 1:** Genotypic values of simulated QTL for a one-locus, two-allele model of gene action when a trait is affected only by additive (second column) and by both additive and dominance (third column).

k	Pure additive(${d}_{i}=0$)	Additive: Dominance
${A}_{1}{A}_{1}$	$2-2p(a)$	$(2-2p)\alpha$
${A}_{1}{A}_{2}$	$1-2p(a)$	$(1-2p)\alpha$
${A}_{2}{A}_{2}$	$-2p\,(a)$	$(-2p)\alpha$

*p*: allelic frequency, *a*: additive effect, *d*_i_: dominance effect, *α* = *a* + *d*(*q* − *p*): average effect of allelic substitution.

**Table 2:** Distribution of simulated QTL effects (Gamma for addtive and normal for epistatic) and corresponding parameters. The dominance QTL effects were derived from additive effects and a degree of dominance derived from a normal distribution.

Genetic Effects	Number of QTL/Interactions	Distribution
additive	300	$G \sim (\alpha =0.42,\beta =8.282)$
dominance	300	${d}_{k}={\delta }_{k}|{\alpha }_{k}|\,,\,{\delta }_{k} \sim N(0.5,1)$
additive × additive	1500	$N \sim (m=0.02,{t}^{2}=0.03)$
additive × dominance	1500	$N \sim (m=0.02,{t}^{2}=0.03)$
dominance × additive	1500	$N \sim (m=0.02,{t}^{2}=0.03)$
dominance × dominance	1500	$N \sim (m=0.02,{t}^{2}=0.03)$

*m*: mean, *t*^2^: variance, *δ*_k_: degree of dominance, *G*~: Gamma distribution, *N*~: normal distribution.

#### Additive and dominance (Ad:Dom)

Dominance arises when the effect of alleles at a locus interact such that the value of heterozygous genotype deviates from the mean value of the homozygous genotypes. The dominance deviation for a particular QTLs was calculated as the difference between the average value of ${A}_{1}{A}_{2}\,\,$genotypes and the mean of ${A}_{1}{A}_{1}$ and ${A}_{2}{A}_{2}$ genotypes. Then, breeding values are $2q[a\,+\,d(q-p)]$ (for ${A}_{1}{A}_{1}$), $(q-p)[a\,+\,d(q-p)]$ (for ${A}_{1}{A}_{2}$) and $-2p[a\,+\,d(q-p)]$ (for ${A}_{2}{A}_{2}$). The dominance deviation at a given QTL locus is the difference between the total genotypic value and the breeding value, and is equal to $-2{q}^{2}d$, $2pqd$ and $-2{p}^{2}d$ for ${A}_{1}{A}_{1}$,$\,{A}_{1}{A}_{2}$ and ${A}_{2}{A}_{2}$, respectively^17^. In this study, the dominance effect QTL *k* was determined as the product of the absolute value of the additive substitution effect and degree of dominance $\,{d}_{k}={\delta }_{k}.|{\alpha }_{k}|$, here,$\,{\delta }_{k}$ is the degree of dominance sampled from a normal distribution with ${\delta }_{k} \sim N(0.5,\,1)$ (Table 2). To create the phenotypic value for individual *i*, a residual ${e}_{i}$ was added to the sum of effects of the true breeding value and of the dominance deviation:

$${y}_{i}=\sum _{k=1}^{nQTL}({X}_{ik}{a}_{k}+{D}_{ik}{d}_{k})+{e}_{i}$$

Above, ${D}_{ik}$ (*i* = 1, …, number of individuals; *k* = 1, …, number of QTLs) is an element of the incidence matrix for dominance genetic effects (${d}_{k})\,\,$with 0, 1, and 0 as entries for ${A}_{2}{A}_{2}$,$\,{A}_{2}{A}_{1},\,\,$and ${A}_{1}{A}_{1}$ genotypes, respectively.

#### Additive, dominance and epistasis (Ad:Dom:Epi)

The simplest quantitative genetic model including epistasis is a two-locus model in which each locus has two alleles. Epistatic gene action influences the average effects of alleles and of dominance deviations, and consequently, the additive and dominance genetic variance^18,19^. In this scenario, we considered the genetic effects on a trait to be due to unlinked QTLs, with additive, dominance and epistatic gene action (Table 3).

**Table 3:** Genotypic values and genotypic frequencies^1^ in a two-locus, two-allele model with additive, dominance, and epistatic gene action.

*A*–*locus genotype*	*f*(*A*_*i*_*A*_*j*_)	*B*–*locus genotype*		
		*B*_1_ *B*_1_	*B*_1_ *B*_2_	*B*_2_ *B*_2_
		*f*(*B*_*k*_*B*_*l*_)		
		${{\boldsymbol{q}}}_{{\bf{1}}}^{{\bf{2}}}$	2*q*_1_*q*_2_	${{\boldsymbol{q}}}_{{\bf{2}}}^{{\bf{2}}}$
${A}_{1}{A}_{1}$	${p}_{1}^{2}$	$\mu +{a}^{A}+{a}^{B}+aa$	$\mu +{a}^{A}+{d}^{B}+ad$	$\mu +{a}^{A}-{a}^{B}-aa$
		${p}_{1}^{2}{q}_{1}^{2}$	$2{p}_{1}^{2}{q}_{1}{q}_{2}$	${p}_{1}^{2}{q}_{2}^{2}$
${A}_{1}{A}_{2}$	$2{p}_{1}{p}_{2}$	$\mu +{d}^{A}+{a}^{B}+da$	$\mu +{d}^{A}+{d}^{B}+dd$	$\mu +{d}^{A}-{a}^{B}-da$
		$2{p}_{1}{p}_{2}{q}_{1}^{2}$	$4{p}_{1}{p}_{2}{q}_{1}{q}_{2}$	$2{p}_{1}{p}_{2}{q}_{2}^{2}$
${A}_{2}{A}_{2}$	${p}_{2}^{2}$	$\mu -{a}^{A}+{a}^{B}-aa$	$\mu -{a}^{A}+{d}^{B}-ad$	$\mu -{a}^{A}-{a}^{B}+aa$
		${p}_{2}^{2}{q}_{1}^{2}$	$2{p}_{2}^{2}{q}_{1}{q}_{2}$	${p}_{2}^{2}{q}_{2}^{2}$

Two locus genotypic frequencies were obtained by multiplication of marginal frequencies under linkage equilibrium^63^.

*μ*: population mean; *a*: additive substitution effect; *d*: dominance deviation;*aa*, *da*, *da* and *dd*: additive × additive, additive × dominance, dominance × additive and dominance × dominance, gene actions respectively; *p* and *q* are major and minor allele frequencies.

Epistasis was simulated only between pairs of QTLs and it included additive × additive (A × A), additive × dominance (A × D), dominance × additive (D × A), and dominance × dominance (D × D) interactions. QTLs were randomly chosen from the 300 QTLs to form 1,500 pairs, and each pair was assigned interaction effects; 1) (A × A) $aa{l}_{k}{l}_{k^{\prime} }$, 2) (A × D) $ad{l}_{k}{l}_{k^{\prime} }$, 3) (D × A) $da{l}_{k}{l}_{k^{\prime} }\,\,$and 4) (D × D) interaction $dd{l}_{k}{l}_{k^{\prime} }$. Here, ${l}_{k}$ and ${l}_{k^{\prime} }$ represent the $k$ and $k^{\prime}$ QTLs. Similar to Wittenburg *et al*.^16^, the epistatic effects were sampled from a normal distribution with parameters shown in Table 2. The phenotype was created by adding ${e}_{i}$ to the sum of simulated additive, dominance and epistatic QTLs effects^20^:

$$\begin{array}{c}{y}_{i}=\sum _{k=1}^{nQTL}{X}_{ij}{a}_{j}+\sum _{k=1}^{nQTL}{D}_{ij}{d}_{j}+\sum _{k=1}^{p-1}\sum _{\begin{array}{c}k^{\prime} =2\\ k^{\prime} \ne k\end{array}}^{p}aa{l}_{k}{l}_{k^{\prime} }+\sum _{k=1}^{p-1}\sum _{\begin{array}{c}k^{\prime} =2\\ k^{\prime} \ne k\end{array}}^{p}ad{l}_{k}{l}_{k^{\prime} }\\ \,\,\,+\sum _{k=1}^{p-1}\sum _{\begin{array}{c}k^{\prime} =2\\ k^{\prime} \ne k\end{array}}^{p}da{l}_{k}{l}_{k^{\prime} }+\sum _{k=1}^{p-1}\sum _{\begin{array}{c}k^{\prime} =2\\ k^{\prime} \ne k\end{array}}^{p}dd{l}_{k}{l}_{k^{\prime} }+{e}_{i}\end{array}$$

Above, $aa{l}_{k}{l}_{k^{\prime} }$,$\,ad{l}_{k}{l}_{k^{\prime} }$,$\,da{l}_{k}{l}_{k^{\prime} }$ and $dd{l}_{k}{l}_{k^{\prime} }$ are the AxA, AxD, DxA, and DxD epistatic effects between QTLs k and k′ (k < k′ = 1, …, p), respectively.

#### Purely epistatic (Epi)

We also simulated a purely epistasic model, without additive and dominance effects at any of the QTLs, as:

$${y}_{i}=\sum _{k=1}^{p-1}\sum _{\begin{array}{c}k^{\prime} =2\\ k^{\prime} \ne k\end{array}}^{p}aa{l}_{k}{l}_{k^{\prime} }+\sum _{k=1}^{p-1}\sum _{\begin{array}{c}k^{\prime} =2\\ k^{\prime} \ne k\end{array}}^{p}ad{l}_{k}{l}_{k^{\prime} }+\sum _{k=1}^{p-1}\sum _{\begin{array}{c}k^{\prime} =2\\ k^{\prime} \ne k\end{array}}^{p}da{l}_{k}{l}_{k^{\prime} }+\sum _{k=1}^{p-1}\sum _{\begin{array}{c}k^{\prime} =2\\ k^{\prime} \ne k\end{array}}^{p}dd{l}_{k}{l}_{k^{\prime} }+{e}_{i}\,$$

The simulation process was carried out in two steps: the QMSim software^21^ was first used to simulate the historical and recent populations and then the outputs were used to design gene action architectures.

#### Genetic variance components

In order to compute genetic variance components based on Cockerham^22^, we assumed that each pairs of QTLs were independent, and the additive and non-additive genetic variances were as in Table 4. Table 5 shows the partition of variance relative to the total variance explained by each source of genetic variation accounted for traits.

**Table 4:** Variance components for main effects (additive and dominance) and two order epistatic interactions that contributed to genetic variance under different genetic architectures.

Additive	${\delta }_{a}^{2}=2pq{[a+d(q-p)]}^{2}=2pq{\alpha }^{2}$
Dominance	${\delta }_{d}^{2}={[2pqd]}^{2}$
Additive × Additive	${\delta }_{aa}^{2}=4\sum {p}_{i}{q}_{k}{(\alpha {\alpha }_{ik})}^{2}$
Additive × Dominance	${\delta }_{ad}^{2}=2\sum {p}_{i}{q}_{k}{q}_{l}{(\alpha {\delta }_{ikl})}^{2}$
Dominanc × Additive	${\delta }_{da}^{2}=2\sum {p}_{i}{p}_{j}{q}_{k}{(\delta {\alpha }_{ikl})}^{2}$
Dominanc × Dominanc	${\delta }_{dd}^{2}=\sum {p}_{i}{p}_{j}{q}_{k}{q}_{l}{(\delta {\delta }_{ijkl})}^{2}$

*a*: additive substitution effect; *d*: dominance deviation; *α*: average allelic effect; *αα*, *αδ*, *δα* and *δδ* are additive × additive, additive × dominance, dominance × additive and dominance × dominance epistatic deviations, respectively; *p* and *q* are major and minor allele frequencies^64^.

**Table 5:** Heritability of simulated traits under various forms of gene action (additive, dominance and epistatic).

Gene action	${{\boldsymbol{h}}}_{{\boldsymbol{a}}}^{{\bf{2}}}$	${{\boldsymbol{h}}}_{{\boldsymbol{d}}}^{{\bf{2}}}$	${{\boldsymbol{h}}}_{{\boldsymbol{a}}{\boldsymbol{:}}{\boldsymbol{a}}}^{{\bf{2}}}$	${{\boldsymbol{h}}}_{{\boldsymbol{a}}{\boldsymbol{:}}{\boldsymbol{d}}}^{{\bf{2}}}$	${{\boldsymbol{h}}}_{{\boldsymbol{d}}{\boldsymbol{:}}{\boldsymbol{a}}}^{{\bf{2}}}$	${{\boldsymbol{h}}}_{{\boldsymbol{d}}{\boldsymbol{:}}{\boldsymbol{d}}}^{{\bf{2}}}$	${{\boldsymbol{H}}}_{{\boldsymbol{b}}{\boldsymbol{r}}{\boldsymbol{o}}{\boldsymbol{a}}{\boldsymbol{d}}\,{\boldsymbol{s}}{\boldsymbol{e}}{\boldsymbol{n}}{\boldsymbol{s}}{\boldsymbol{e}}}^{{\bf{2}}}$
Purely Additive	**0.30**	0.00	0.00	0.00	0.00	0.00	0.30
Additive:Dominance	**0.30**	**0.10**	0.00	0.00	0.00	0.00	0.40
Additive:Dominance:Epistatic	**0.30**	**0.10**	**0.10**	**0.10**	**0.10**	**0.10**	0.80
Purely Epistatic	0.00	0.00	**0.10**	**0.05**	**0.05**	**0.10**	0.30

${h}_{a}^{2}$: additive heritability, ${h}_{d}^{2}$: dominance heritability, and ${h}_{a:a}^{2}$, ${h}_{a:d}^{2}$, ${h}_{d:a}^{2}$, and ${h}_{d:d}^{2}$ are additive by additive, additive by dominance, dominance by additive, and dominance by dominance epistatic heritabilites, respectively.

### Real Data

The data set consisted of records on 1,351 broiler chickens provided by Aviagen Ltd (Aviagen Ltd, Newbridge, UK) for three traits: body weight (BW), ultrasound of breast muscle at 35 days of age (BM), and hen-house egg production (HHP) defined as the total number of eggs laid between weeks 28 and 54 per bird. Phenotypic records for BW and BM were pre-corrected for a combined effect of sex (525 males and 826 females), hatch week, contemporary group of parents and pen in the growing farm, whereas phenotypic records for HHP were pre-adjusted for hatch effects. All individuals were genotyped with a 600 K Affymetrix SNP chip (Affymetrix, Inc., Santa Clara, CA, USA). More precisely, 580,954 SNP genotypes were available in the dataset. Markers with MAF $<$ 1% were removed and missing genotypes for the remaining SNPs were imputed using the Beagle software^23^. All SNPs were subsequently kept if they presented a genotype call rate >95% and were in Hardy–Weinberg equilibrium. Individuals were kept if their genotype call rate >95%. Deviation from Hardy–Weinberg equilibrium was assessed by the Pearson’s chi-square test with a significance threshold of 10^− 6^. After edits, 354,364 autosomal SNPs remained for the analysis. Mean MAF was equal to 0.27. Only SNPs on 28 chromosomes were considered, covering 919 Mb of the *Gallus gallus* genome. The PLINK software^24^ was used to edit the data.

#### Genome-assisted prediction model

The performance of 14 different prediction models that differ with respect to assumptions regarding distribution of marker effects was evaluated. The parametric models included GBLUP^25,26^, ridge regression BLUP (rrBLUP)^27,28^, the least absolute shrinkage and selection operator (LASSO)^29,30^, the elastic net (EN)^31^, Bayesian ridge regression (BRR)^5,31,32^, BL^33^, BayesA^27,34^, BayesB^27,34^, and BayesC^27,34^. In addition, the following non-parametric models were evaluated: reproducing kernel Hilbert space regression (RKHS)^35–37^, SVM^38^, relevance vector machine (RVM)^39^ and Gaussian Processes (GP)^39,40^ and random forest (RF). Although GBLUP and the GP use similar approaches, GP which is often used in machine learning, predict the value for an unseen point from training data and defined as a collection of random variables^40,41^.

To implement the BayesA, BayesB, BayesC, BRR, BL, and RKHS, we used BGLR R package developed by Pérez and de los Campos^42^ and the glmnet function from the glmnet R-package were used for LASSO and EN^43^. The rvm, ksvm functions from the kernlab package^44^ were used to predict genomic breeding values for RVM, SVM, and GP. In addition, we used the mixed.solve function from rrBLUP package^28^ to perform GBLUP and rrBLUP and the randomForest option from the e1071 package^45^ for RF.

To compare the performance of the different prediction models, we used 20 replicates of a five-fold cross-validation scheme as described in Pérez-Cabal *et al*.^46^. The data were divided into training (80%) and testing (20%) sets. The training set was used to fit the models and the testing set to measure performance of the prediction models. The procedure was repeated 20 times at random, yielding 100 cross-validation runs.

For each cross-validation scenario, three criteria were measured: (i) predictive accuracy defined as the correlation between phenotypic values and the predicted genomic values ($${r}_{y,GEBV})$$, (ii) the “empirical” accuracy defined as the correlation between true breeding values (TBV) and predicted genomic breeding values ($${r}_{TBV,GEBV}$$) (because of unknown TBV, this criterion was not used in the chicken data set) and, (iii) a test for empirical prediction bias done by regressing phenotypes (simulated and real) on the GEBVs.

### Availability of data and materials

The datasets generated and/or analyzed during the current study are not publicly available due to the Aviagen Ltd (Aviagen Ltd, Newbridge, UK) polices.

### Ethical approval and consent to participate

The article does not contain any studies with human subjects performed by the authors. The data analysis was conducted in the Department of Animal Science at the University of Wisconsin-Madison, U.S.A.

## Results and Discussion

### Predictive accuracy and empirical accuracy of genomic predictions

**Figure 1:**
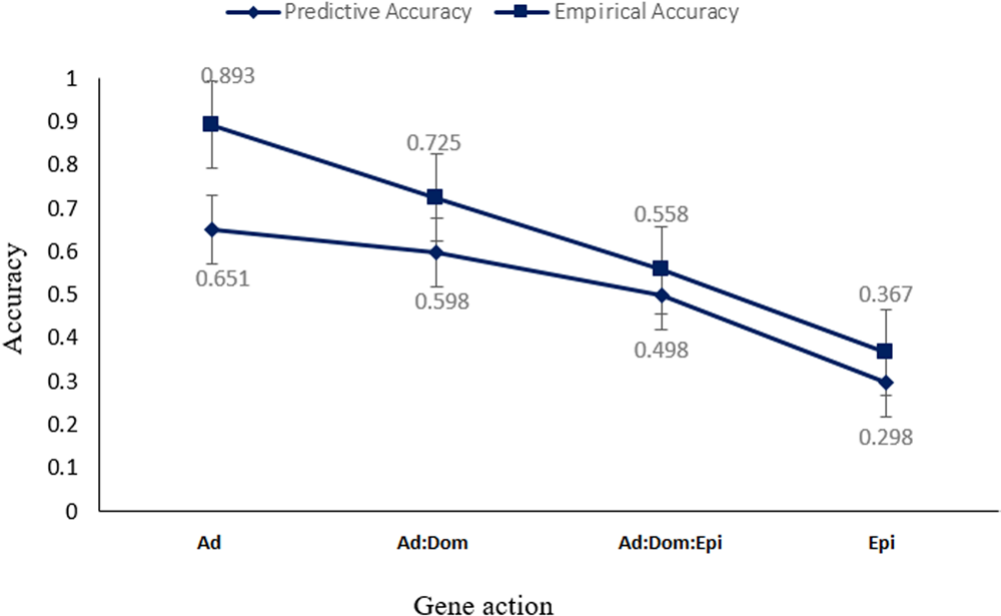
Overall mean (standard error) of predictive and empirical accuracy of different prediction models under various gene action scenarios: purely additive (Ad), additive and dominance (Ad:Dom), additive dominance and epistasis (Ad:Dom:Epi), and pure epistasis (Epi).

Figure 1 shows the mean and standard errors (the 100 cross-validation values) of predictive and empirical accuracy over all prediction models. Prediction accuracies decreased when gene action was more complex, although the two extreme architectures (i.e. Ad and Epi) had the same broad sense heritability (${H}^{2}$= 0.30). The largest difference between predictive and empirical accuracy was under the Ad scenario. This may be due to the fact that the additive model was the simplest, so the prediction task is less challenging to the models.

**Figure 2:**
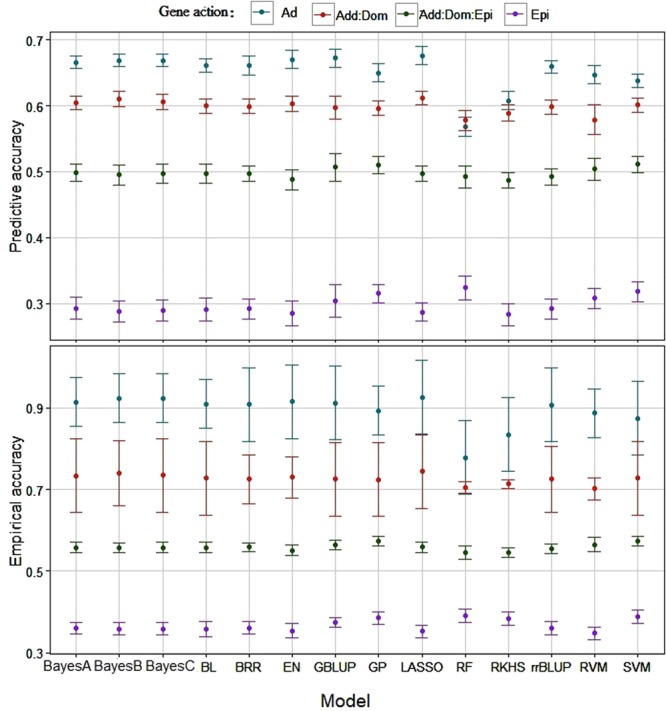
Predictive and empirical accuracies of genomic prediction models for traits simulated under purely additive (Ad), additive:dominance (Add:Dom), additive:dominance:epistatic (Add:Dom:Epi), and purely epistatic (Epi) gene action scenarios with a broad sense heritability of 0.30, 0.40, 0.80 and 0.30, respectively. Prediction models: BayesA, BayesB BayesC, Bayesian least absolute shrinkage and selector operator (BL), Bayesian ridge regression (BRR), elastic net (EN), genomic best linear unbiased prediction (GBLUP), Gaussian process (GP), least absolute shrinkage and selector operator (LASSO), random forest (RF), reproducing kernel Hilbert spaces regression (RKHS), ridge regression best linear unbiased prediction (rrBLUP), relevance vector machine (RVM), and support vector machine (SVM).

Predictive and empirical accuracies of prediction models for traits simulated under Ad, Add:Dom, Add:Dom:Epi, and Epi gene actions are depicted in Fig. 2. Both measures of accuracy showed the same trend across gene action scenarios. The highest predictive and empirical accuracies were consistently obtained under Ad (0.56 and 0.90, respectively), in which genetic values of individuals were only influenced by additive QTL effects. Accuracy decreased as genetic complexity increased (0.33 and 0.4 for Epi). The results show QTL gene action affects empirical and predictive accuracies in genomic prediction. Our findings under a purely additive scenario are in agreement with Daetwyler *et al*.^47^. They compared two parametric models (GBLUP and BayesB) using data with three different effective population sizes coupled with a wide range of number of additive QTLs. They found that GBLUP had a stable accuracy, whereas BayesB slightly outperformed GBLUP when the number of QTLs was small. A similar finding was reported by Clark *et al*.^48^, who investigated the effect of genetic architecture on predictive performance of rrBLUP and BayesB. In this study, BayesB outperformed rrBLUP if the trait to be predicted was influenced by a few rare QTLs with a large effect. However, the previous studies did not examine non-parametric models or genetic architectures other than the additive gene action.

Predictive and empirical accuracies did not differ among prediction models at any of the gene action scenarios, except for RF and RKHS, which produced the lowest performance when predicting the trait under Ad genetic architecture but slightly outperforming the other prediction models under Epi. Although parametric models differ in prior assumptions made about marker effects^49^, their predictive ability was similar and they globally obtained higher accuracies, especially under Ad genetic architecture.

Among parametric models, LASSO and GBLUP yielded the highest accuracy of prediction when only additive genetic effect influenced the phenotype. Conversely, non-parametric models such as RKHS, delivered better predictive performance when non-additive effects were present. This is because non-parametric or semi-parametric models can build (co)variance structures capable of capturing more complex modes of gene action than linear smoothers^50^. Our results are in agreement with previous studies; for example Howard *et al*.^12^ reported that parametric models predicted phenotypic values worse when the underlying architecture was entirely epistatic, whereas parametric models produced slightly better predictions than non-parametric models when additively assumptions held. Further, parametric genome-based prediction models were unable to predict chill coma recovery, an adaptive trait in Drosophila. Previous whole genome scan suggested that this trait exhibited epistatic interactions involving many loci^2^. Possibly, non-parametric models account better non-additive effects while making weaker assumptions^51^. Thus, non-parametric regression models seem to be well-suited for modeling such traits.

Differences in predictive ability among non-parametric models could be due to the intrinsic ways in which marker information is incorporated by various prediction models. While models make no assumptions about gene action, non-linearity is introduced in specific ways^52^. For instance, RKHS with a single Gaussian kernel may yield different results compared to a multi-kernel specification e.g.,^53^. Further, the differences among parametric models when a specific genetic architecture was assumed, may be due to difference in the ability of the prediction models in capturing linkage disequilibrium between markers and QTLs leading to different prediction accuracies^49,54^.

Arguably, a higher genomic heritability results in genetic values that perform better at predicting yet-to-be observed phenotypes. For example, prediction accuracies for wheat resistance to yellow and stem rust was related to their lower and highest heritability, respectively^55^. Similar results were found for grain yield (low heritability) versus grain moisture (high heritability) in maize, with the respective accuracies of prediction at 0.58 and 0.90^56^. Nevertheless, predictive ability does not depend on heritability only. For instance, prediction accuracy for flour protein content (heritability = 0.56) and sucrose solvent retention (heritability = 0.45) was 0.64 and 0.74, respectively, in double-haploid biparental wheat lines^57^. As shown in our simulation study, accuracy of genomic prediction was sensitive not only with respect to heritability of a trait but also with respect to gene action.

### Prediction bias

**Figure 3:**
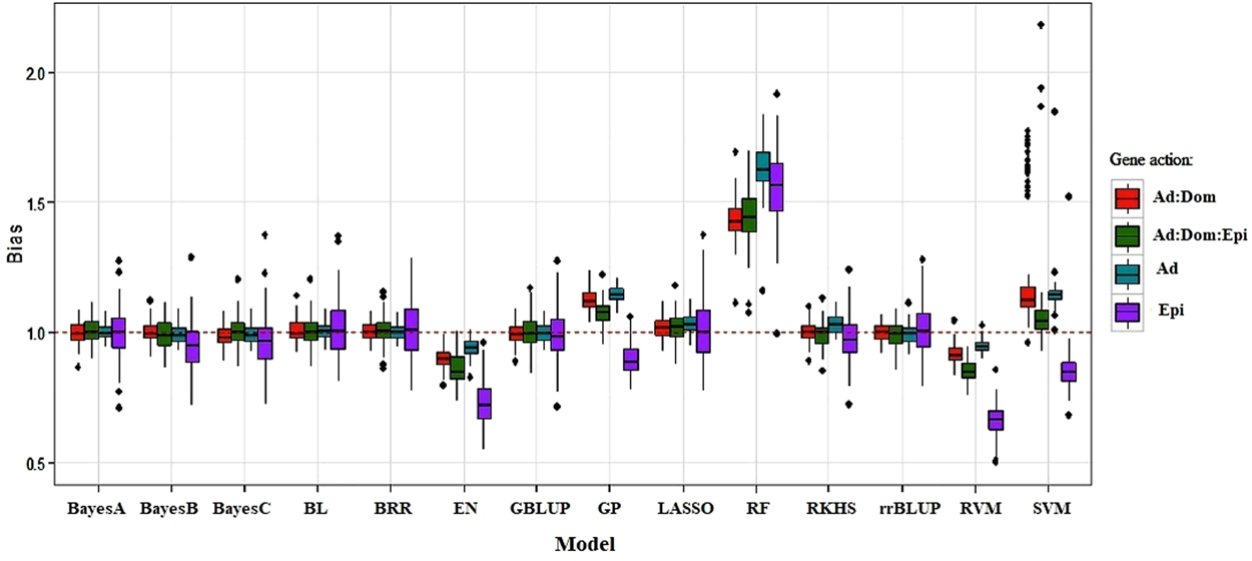
Boxplots of bias (regression coefficient of simulated phenotypes on genomic estimated breeding values) for traits simulated under purely additive (Ad), additive:dominance (Ad:Dom), additive:dominance:epistatic (Ad:Dom:Epi) and pure epistatic (Epi) gene action scenarios and heritability of 0.30, 0.40, 0.80 and 0.30, respectively. Prediction models: BayesA, BayesB, BayesC, Bayesian least absolute shrinkage and selector operator (BL), Bayesian ridge regression (BRR), elastic net (EN), genomic best linear unbiased prediction (GBLUP), Gaussian process (GP), least absolute shrinkage and selector operator (LASSO), random forest (RF), reproducing kernel Hilbert spaces regression (RKHS), ridge regression best linear unbiased prediction (rrBLUP), relevance vector machine (RVM), and support vector machine (SVM). Outliers are denoted as black dots.

Figure 3 shows boxplots of the regression of simulated phenotypes on the predicted genomic values. “Unbiased prediction models” are expected to have a regression with a small intercept and a slope equal to 1 (red dashed horizontal line in Fig. 3); the regression coefficients greater than 1 indicate under-prediction and smaller than 1 indicate an over-statement prediction^30^. BayesA, BayesB, BayesC, BL, BRR, GBLUP, RKHS, and, rrBLUP produced nearly unbiased predictions, irrespectively of the genetic architecture underlying the trait. EN and RF systematically over and under predicted genetic architecture scenarios, respectively. GP and SVM over predicted the trait under Epi architecture, and under predicted otherwise. Genetic architecture of the trait had a great influence on predictive ability of the models tested. Less biased, more precise, and stable prediction models should be preferred. Our results indicate that an inadequate representation of genetic architecture may lead to biased predictions when genomic data are used as inputs. In such situations, appropriate prediction models that are more capable to capture genetic architecture of complex traits for correcting the bias of predictions are required^58,59^.

### Hierarchical clustering of predicted genetic values

A hierarchical clustering algorithm “Ward’s method”^60^ was applied to compute a distance matrix from three sources (predictive and empirical accuracies, and bias) for all implemented prediction models. The solution obtained with Ward’s method was refined using the *k*-means algorithm taking an agglomerative approach or bottoms up approach^61^ so that it starts with own cluster and each pairs of clusters were merged together as one moves up the hierarchy^62^.

**Figure 4:**
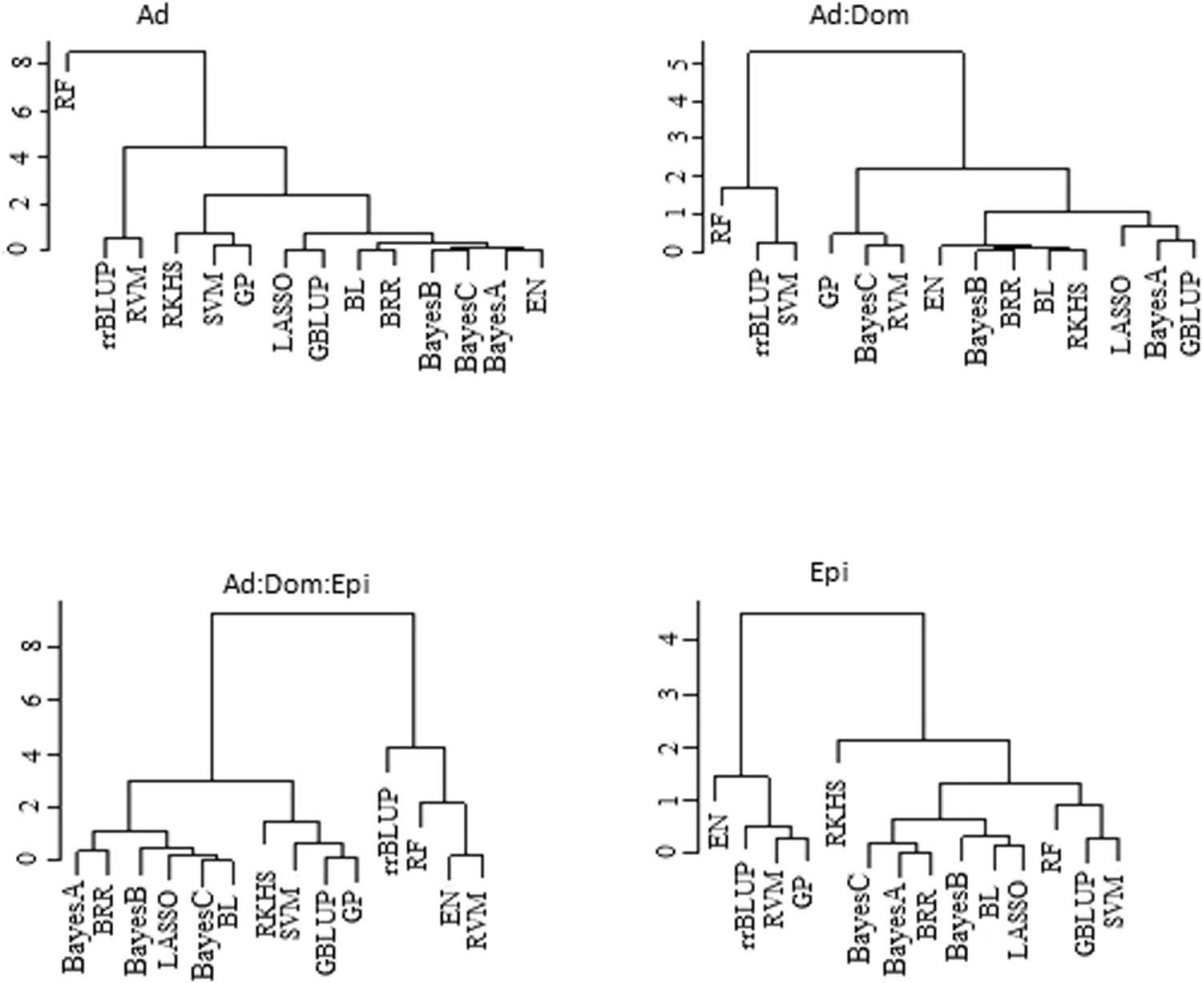
Ward’s hierarchical clustering on predicted genomic values derived from traits simulated under purely additive (Ad), additive:dominance (Ad:Dom), additive:dominance:epistatic (Ad:Dom:Epi) and purely epistatic (Epi) gene action. Prediction models: Bayes A, Bayes B, Bayes C, Bayesian least absolute shrinkage and selector operator (BL), Bayesian ridge regression (BRR), elastic net (EN), genomic best linear unbiased prediction (GBLUP), Gaussian processor (GP), least absolute shrinkage and selector operator (LASSO), random forest (RF), reproducing kernel Hilbert spaces regression (RKHS), ridge regression best linear unbiased prediction (rrBLUP), relevance vector machine (RVM) and support vector machine (SVM).

Results (Fig. 4) showed that under Ad gene action, parametric and non-parametric models (notably RF, GP, SVM, and RKHS) were grouped into different clusters. In, the Ad:Dom model, the dendrogram showed a slightly different structure; for example, BayesC was placed together with GP, and RVM, and RKHS were placed within a parametric group. When epistatic interaction effects were included (Ad:Dom:Epi, and Epi), all Bayesian models and LASSO settled in the same category. For Ad:Dom:Epi, RKHS, SVM, GBLUP, and GP were grouped together, all Bayesian models were grouped in separate cluster, and RVM and RF were in the same cluster with rrBLUP and EN. Within the Epi architecture, RKHS regression was separated from all other models, and some non-parametric models were allocated to groups that combine parametric models. In summary, the dendrogram topology did not separate non-parametric from parametric models clearly, when gene action was not additive.

### Chicken dataset

**Figure 5:**
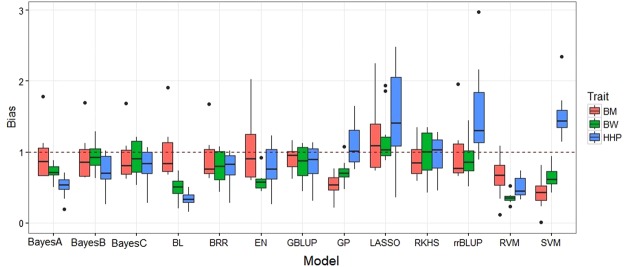
Boxplots of bias (regression coefficient of observed phenotypes on genomic estimated breeding values) obtained in the testing sets from a 20-fold cross validation using chicken data for body weight (BW), breast meat (BM) and hen-house production (HHP). Prediction models: Bayes A, Bayes B, Bayes C, Bayesian least absolute shrinkage and selector operator (BL), Bayesian ridge regression (BRR), elastic net (EN), genomic best linear unbiased prediction (GBLUP), Gaussian process (GP), least absolute shrinkage and selector operator (LASSO), random forest (RF), reproducing kernel Hilbert spaces regression (RKHS), ridge regression best linear unbiased prediction (rrBLUP), relevance vector machine (RVM) and support vector machine (SVM). Outliers are denoted as black dots.

The results obtained with chicken data on predictive accuracy and bias indicated that GP and GBLUP consistently produced the least biased, most precise, and most stable estimates of predictive accuracy for HHP and BM (Fig. 5 and Table 6). For BW, BayesA and BayesB, and LASSO yielded the highest predictive accuracies, and LASSO was at least as good as or ever better than BayesA and BayesB in terms of unbiasedness. RKHS performed best the among non-parametric models. Other prediction models performed inconsistently across the traits and suffered varying degrees of over- or under-prediction and numerical instability. In general, all models tended more to over predict yet-to-be observed phenotypes than to under predict, whereas in the simulations, most models tended to under predict measured phenotypes.

**Table 6:** Average correlations between phenotypes and predicted breeding values obtained in the testing sets from a 20-fold cross validation using the chicken data for body weight (BW), breast meat (BM), and hen-house production (HHP).

Models	Traits		
	BW	BM	HHP
BayesA	0.320 (0.023)	0.195 (0.012)	0.209 (0.017)
BayesB	0.330 (0.034)	0.196 (0.012)	0.219 (0.017)
BayesC	0.188 (0.023)	0.190 (0.012)	0.220 (0.017)
BL	0.196 (0.023)	0.188 (0.012)	0.186 (0.016)
BRR	0.190 (0.024)	0.176 (0.011)	0.247 (0.018)
EN	0.249 (0.027)	0.198 (0.015)	0.231 (0.019)
GBLUP	0.192 (0.021)	0.268 (0.009)	0.221 (0.017)
GP	0.178 (0.023)	0.140 (0.011)	0.227 (0.017)
LASSO	0.284 (0.019)	0.201 (0.015)	0.176 (0.010)
RKHS	0.191 (0.018)	0.206 (0.009)	0.219 (0.016)
rrBLUP	0.175 (0.016)	0.169 (0.010)	0.236 (0.015)
RVM	0.185 (0.026)	0.159 (0.024)	0.196 (0.015)
SVM	0.172 (0.024)	0.161 (0.018)	0.202 (0.017)

Prediction models: BayesA, BayesB, BayesC, Bayesian least absolute shrinkage and selector operator (BL), Bayesian ridge regression (BRR), elastic net (EN), genomic best linear unbiased prediction (GBLUP), Gaussian processor (GP), least absolute shrinkage and selector operator (LASSO), reproducing kernel Hilbert spaces regression (RKHS), ridge regression best linear unbiased prediction (rrBLUP), relevance vector machine (RVM), and support vector machine (SVM).

Results obtained with the chicken data also show that the performance of the prediction models was trait dependent. Our results support the view that there are no universally best prediction models and that prediction performance is not necessarily indicating mode of gene action.

## Conclusions

This study compared nine parametric and five non-parametric genome-based prediction models with simulated and real data sets. Our study confirms that when gene action was additive, parametric models provide better prediction than non-parametric models. Conversely, some of the non-parametric models produced a better performance when epistatic interaction effects underlie phenotypic variation. For example, GP, RKHS, and RF models, which exploit a non-linear relationship between SNP markers and phenotypes, delivered a higher predictive accuracy and a smaller bias of prediction under epistatic gene action.

Assumptions and treatment of marker effects are two main factors that affect predictive abilities of a prediction models. If non-additive genetic effects are important, genome-based tools can be used to identify the nature and components of interacting genetic systems, and perhaps genomic prediction schemes can be designed to exploit non-additive genetic sources of variation.
